# Novel Immunotherapeutic Approaches to Target Alpha-Synuclein and Related Neuroinflammation in Parkinson’s Disease

**DOI:** 10.3390/cells8020105

**Published:** 2019-01-31

**Authors:** Maria Angela Samis Zella, Judith Metzdorf, Friederike Ostendorf, Fabian Maass, Siegfried Muhlack, Ralf Gold, Aiden Haghikia, Lars Tönges

**Affiliations:** 1 Department of Neurology, St. Josef-Hospital, Ruhr-University Bochum, 44791 Bochum, Germany; maria.zella@rub.de (M.A.S.Z.); judith.metzdorf@rub.de (J.M.); friederike.ostendorf@googlemail.com (F.O.); siegfried.muhlack@rub.de (S.M.); ralf.gold@rub.de (R.G.); aiden.haghikia@rub.de (A.H.); 2 Department of Neurology, St. Josef- Hospital, Katholische Kliniken Ruhrhalbinsel, Contilia Gruppe, 45257 Essen, Germany; 3 Department of Neurology, University Medical Center, 37075 Goettingen, Germany; fabian.maass@med.uni-goettingen.de; 4 Neurodegeneration Research, Protein Research Unit Ruhr (PURE), Ruhr University Bochum, 44801 Bochum, Germany

**Keywords:** Parkinson’s disease, neuroinflammation, alpha-Synuclein, immunotherapy

## Abstract

The etiology of Parkinson’s disease (PD) is significantly influenced by disease-causing changes in the protein alpha-Synuclein (aSyn). It can trigger and promote intracellular stress and thereby impair the function of dopaminergic neurons. However, these damage mechanisms do not only extend to neuronal cells, but also affect most glial cell populations, such as astroglia and microglia, but also T lymphocytes, which can no longer maintain the homeostatic CNS milieu because they produce neuroinflammatory responses to aSyn pathology. Through precise neuropathological examination, molecular characterization of biomaterials, and the use of PET technology, it has been clearly demonstrated that neuroinflammation is involved in human PD. In this review, we provide an in-depth overview of the pathomechanisms that aSyn elicits in models of disease and focus on the affected glial cell and lymphocyte populations and their interaction with pathogenic aSyn species. The interplay between aSyn and glial cells is analyzed both in the basic research setting and in the context of human neuropathology. Ultimately, a strong rationale builds up to therapeutically reduce the burden of pathological aSyn in the CNS. The current antibody-based approaches to lower the amount of aSyn and thereby alleviate neuroinflammatory responses is finally discussed as novel therapeutic strategies for PD.

## 1. Introduction

Parkinson’s disease (PD) is the second-most common neurodegenerative disorder and affects 2–3% of the general population with an age of more than 65 years. It is characterized by a multitude of pathophysiological alterations that are linked to the dysfunctional state of the protein alpha-Synuclein (aSyn). In their typical clinical presentation, PD patients exhibit deficits in motor function with bradykinesia, rigidity, tremor, and possibly postural instability, reflecting the degeneration of dopamine processing neurons in the substantia nigra pars compacta and alterations of dopaminergic neurotransmission to basal ganglia motor circuits [1,2,3]. Apart from the motor symptoms, there are substantial impairments in non-motor domains, including signs of cognitive deficits, psychiatric comorbidities, daytime sleepiness, or autonomic failure [4]. The wide variety of symptoms clearly indicates that many different brain regions and neurotransmitter systems are affected [5]. As a causal pathological agent, aSyn has been identified, but the entirety of disease processes cannot be attributed to the dysfunction of a single protein [6]. After intensive research over the last decades, it is now generally accepted that aSyn pathology is tightly associated with CNS neuroinflammation in PD. The evidence for this phenomenon extends over basic science studies in cell culture or animal models to increasing data by human examinations, which have been collected post mortem but also in vivo [7]. However, the importance of neuroinflammation for the development and progression of the PD is still controversial [8,9,10]. In order to weigh the scientific findings, clearly identified pathomechanisms in humans should play a central role in further analysis. This is of particular importance considering the new immunological therapeutic approaches currently being tested in human clinical trials. In vitro and in vivo animal models of PD serve to evaluate and differentiate human pathophysiological findings. In addition, they enable us to design targeted immunological therapies for human use. 

In this review article, we present the current state of basic research on the role of aSyn and its direct lesioning effects on neuronal cells. The impact of aSyn as an aggravator but also a potential facilitator [11] for neuroinflammation is a strong focus of this review, and will be analyzed based on cell types involved in neuroinflammation. The interactions of aSyn with astroglia, monocytes and microglia, and T lymphocytes are depicted and resulting cellular pathologies and their associated pathomechanisms are discussed. These findings are related to human neuropathological data and analyzed for their significance, but also plausibility. Finally, a clear rationale emerges that reducing the burden of aSyn pathology is a promising approach for a disease modifying therapy of PD. The preclinical results and designs of current human studies employing anti-aSyn directed antibodies as immunotherapy for PD will be presented and evaluated in a look-ahead for future developments. 

## 2. Alpha-Synuclein as Direct Stressor for CNS Neurons

ASyn is a soluble cytoplasmic protein with a length of 140 aa (amino acids) being encoded in the *SNCA* gene. Its primary structure can be subclassified into three regions: an amphipathic N-terminal domain (1–61 aa), a hydrophobic domain (61–95 aa) with the non-amyloid-beta component (NAC), and the N-terminal domain (96–140 aa), which is highly acidic [12,13,14]. ASyn is expressed throughout the CNS, but can also be found in the PNS and other tissues like red blood cells [15]. ASyn aggregates that are localized in so-called Lewy bodies are a neuropathological hallmark of human PD [1] and point mutations in the *SNCA* gene cause familial forms [14].

The physiological role of aSyn is only incompletely understood, but diverse studies have shown that it plays an important role in synaptic plasticity and neurotransmitter release [12,15,16]. ASyn is stored in the presynaptic terminal, where it presumably interacts with the soluble N-ethylmaleimide sensitive factor (NSF) attachment receptor (SNARE) complex, whose circle of assembly and disassembly is important for the continuing release of neurotransmitters. A direct interaction with synaptobrevin-2/vesicle-associated membrane protein 2 and phospholipids seems to promote the assembly of the SNARE-complexes [13,17], and over-expression of aSyn leads to an impairment of different synaptic functions, like vesicle trafficking and recycling [18,19]. 

Under physiological conditions, native aSyn appears as a soluble monomer, but in cases of oxidative stress or a change in pH level, it aggregates to insoluble fibrils, which have a β-sheet conformation. It is able to organize itself in oligomers and amyloid fibrils as aggregates that can harm different cell organelles, like the Golgi apparatus or mitochondria, and then affect mechanisms like synaptic vesicle release [14]. An interesting feature of aSyn is its propagation mechanism, as there is increasing evidence that aSyn is transmitted between neurons. After transport of aSyn assemblies along the axons, it is released in the extracellular space and can then be uptaken by another neuron [16]. Because of the demonstrated spread of aSyn, it is considered by some authors to have a prion-like behavior [20], although this hypothesis is heavily debated [21,22]. Interestingly, Yamada et al. demonstrated recently that the release of aSyn in vivo is also regulated by neuronal activity, as well as by extrinsic mechanisms and stressors [23].

Extracellular aSyn seems to have deleterious effects on neighboring neurons and glia. Various in vitro studies have been performed to demonstrate direct effects on neuronal cells. Exogenous fibrils, which were internalized by primary hippocampal neurons, induced pathological misfolding in endogenous aSyn. This led to phosphorylation, ubiquitination, and finally aSyn aggregation. Interestingly, there was no need for an overexpression of wild-type aSyn because the presence of endogenous aSyn was sufficient [24,25]. Hassink et al. used primary cortical neurons from rats to evaluate the effect of exogenous aSyn. After exposition to extracellular aSyn, they observed an increased uptake of aSyn, followed by intracellular formations of aSyn assemblies. At the same time, a spontaneous initial increase of synaptic activity was detected, which later reversed and resulted in overall activity reduction [26]. Importantly, the impairment of cellular functions by aggregated aSyn is not limited to dopaminergic neuronal cells. Long-term potentiation in hippocampal brain slices is negatively impacted by extracellular aSyn, supposedly by a temporary inhibition of NMDA receptors [14,27]. Direct injections of disaggregated aSyn in the substantia nigra of wild-type rats caused neuronal cell death and behavioral motor deficits [28]. Even after intravenous injection, aSyn crosses the blood brain barrier [28], or is found in the contralateral hemisphere of wild type mice following previous injection of aSyn into the ipsilateral striatum [29]. Virus-mediated overexpression of wildtype or transgenic aSyn injected into the mouse substantia nigra can lead to loss of nigral dopaminergic cells and of their axons, and additionally impairs the intrinsic regenerative capacity of dopaminergic axons [30].

These data clearly demonstrate direct and often deleterious effects of both soluble and aggregated aSyn on neuronal cell populations, including dopaminergic neurons. However, extracellular aSyn also activates glial cells and provokes a multitude of immunological reactions, which can be classified under the term neuroinflammation.

## 3. Alpha-Synuclein as Promotor of Neuroinflammation

With more than 50% of all cells in the brain, glial cells form the largest cell population and thus comprise a number of approximately 1 trillion [31]. In the CNS, they mainly differentiate into astroglia, microglia, and oligodendroglia, which are all susceptible to aSyn and may react with a neuroinflammatory response. Whether a glial cell activation is deleterious or even beneficial for the CNS milieu and its neurons is often hard to say and strongly depends on the specific context. However, in order to develop an interfering strategy to alleviate aSyn-triggered mechanisms, the cellular responses of glia must be well known. However, with respect to neuroinflammation, T lymphocytes and their interaction with microglia also seem to play an important role in PD. Here, we present the most relevant findings from cell-based and animal studies, differentiated according to the three most important cell populations involved.

### 3.1. Astroglia

Astroglia are essential for the proper functioning of CNS neurons. They provide structural and metabolic cerebral homeostasis, regulate water transport, blood flow and synaptic transmission, and produce neurotrophic factors. Thus, their role of a more passive cell population, initially assumed to be rather insignificant, was significantly expanded. In addition, there is a close interaction with microglial and oligodendroglial cells [32]. 

There is only little experimental data on aSyn-mediated stimulation of astroglia available. In cell culture, aSyn was a strong TLR-4 mediated stimulation factor for astrocytes and was taken up by these via endocytosis [33], so that it can be assumed that astrocytes contribute significantly to the clearance and degradation of extracellular aSyn. In the case of astrocyte-selective overexpression of the aSyn A53T mutant, it accumulated significantly and led to reactive astrogliosis associated with cerebral microhemorrhages and reduction of astroglial glutamate transporters [34]. On the other hand, transgenic model systems were used and also demonstrated an important neuroprotective potential of astrocytes. Selective overexpression of the transcription factor Nrf2 in astrocytes in transgenic aSyn-A53T mutant mice delayed the onset of clinical disease and significantly prolonged survival. Furthermore, the amount of aSyn-A53T protein aggregates in mouse brain was significantly reduced and dysfunctional protein degradation, including chaperone-mediated autophagy and macroautophagy, was reduced. Interestingly, the survival of motoneurons was also improved and gliosis and oxidative stress in the spinal cord were reduced, so that non-dopaminergic cells and the CNS tissue structure in general benefited from the modified astrocyte function [35]. 

Astrocytes can absorb extracellular aSyn from neurons and degrade it into lysosomal compartments [36]. Studies on human astrocytes derived from embryonic stem cells have shown that aggregated aSyn can be intercellularly exchanged between astrocytes via small tubular “tunneling nanotubes” (TNT) to avoid overloading of individual cells. In return, the endangered astrocytes received energy-providing mitochondria from the healthier cells [37]. 

It can be concluded that astrocytes play an important modulating role in the progression of PD neuropathology, not least due to their very high abundance and their buffering effect, e.g., for spreading of aSyn. In the case of overloading with aSyn, there is a risk of decompensation with consecutive pro-inflammatory effects, activation of microglia, and collapse of the homeostasis in the CNS. 

### 3.2. Microglia

Microglial cells are a central part of the innate immune system of the CNS and account for up to 20% of the total glial population [38]. The physiological role of resident microglia in the CNS comprises the preservation of homeostasis by continuous monitoring of the local milieu, and if necessary, phagocytose or pinocytose degradation products or cellular residues [39,40]. In this case, the microglia present morphologically with strongly ramified and highly mobile cell processes that are in close contact with other glial and neuronal cells [41,42]. Different stimuli, such as neuronal cell death, mechanical stress, infectious agents, or cellular toxins, can drive microglia to a transformation to an altered morphological phenotype. This induces a polarization of microglia into a proinflammatory neurotoxic or anti-inflammatory neuroprotective phenotype, traditionally referred to as M1 or M2 type. For example, proinflammatory M1 microglia are characterized by the secretion of factors, such as NO, ROS, IL-1b, IL-6, or TNF-α, whereas anti-inflammatory M2 microglia secrete IL-4, IL-10, IL-13, or TGF-β [43,44]. The functional phenotype of the reactive microglia strongly depends on distinct stimulating factors at the beginning or during the progression of PD, and on the respective localization in the CNS [45,46], so that the M1/M2 concept for the differentiated function of the microglia seems to be rather simplified [47]. 

Constitutive transgenic overexpression or local viral-induced overexpression of aSyn results in an increased number of microglial cells in the SN [48,49]. Transgenic mice overexpressing aSyn also showed a distinct microglial activation in the striatum from the first month of life, and only then in the SN from the fifth month of life persisting until the 14th month of life. This was accompanied by increased levels of TNF-α in the striatum and increased levels of TNF-α, TLR1, TLR4, and TLR8 in the SN [50]. Extracellular aSyn released from neuronal cells was demonstrated in an elegant study using in silico, in vitro, and in vivo approaches to be an endogenous agonist for Toll-like receptor 2 (TLR2), which activates inflammatory responses in microglia [51]. 

In vitro, numerous experiments have shown that the aSyn wildtype or mutant variants activate microglia and can pathologically increase microglial inflammatory signals in an autocrine or paracrine manner [7]. In addition to activation of ROS, matrix metalloproteinases or activation of the NFB/AP-1/Nrf2 pathway play an important role [52,53]. Williams et al. investigated the involvement of microglial MHCII complex in alpha-synuclein-induced neurodegeneration. By knock-out of the class II transactivator (CIITA), they inhibited MHCII expression on primary microglia. Stimulation with fibrillar aSyn in vitro then led only to a reduced inflammatory response with less iNOS expression and antigen processing. In vivo, RNA silencing of CIITA led to a reduced MHCII expression, less peripheral immune cell infiltration into the CNS, and reduced aSyn-induced neurodegeneration [54]. In a recent study, a possible pathogenetic contribution of the intestinal microbiome was investigated in mice overexpressing aSyn. It was shown that the microbiome is essential for the development of motor deficits, aSyn aggregation, and microglia activation. Short-chain fatty acids were identified as modulators of microglial activation [55].

Beside this inflammatory response, microglia cells are directly involved in the clearance mechanism of aSyn [56]. In investigating the phagocytic activity of BV2 microglial cells after incubation with aSyn in different aggregation states, an increased internalization of fibrillar aSyn was observed [57]. Another study showed high uptake of aSyn by primary microglia, independent of its aggregation, whereas the C-terminally truncated form of aSyn had the strongest effect on inflammation and oxidative stress, with the TLR4 pathway being critically involved [58]. Besides the aggregation state, PD related mutations of aSyn influence the phagocytic activity of microglia. For the wildtype and A53T mutant form, an enhanced phagocytosis by primary microglia was observed, whilst incubation with the A30P or E46K mutant showed no effect [59]. Interestingly, concentration levels of aSyn also affect the cellular clearance capacity. In an elegant study with induced Pluripotent Stem Cell (PSC) lines generated from early onset PD patients with *SNCA* A53T and *SNCA* triplication mutations, it was shown that PSC-macrophages have a significantly reduced phagocytosis capability with the *SNCA* triplication. This could be phenocopied by adding monomeric aSyn to the cell culture medium. While the PSC-macrophages were, in principle, capable of clearing aSyn, their ability was compromised by high levels of exogenous or endogenous aSyn [60]. 

Overall, it can be stated that the microglia detect damaging events in the CNS very sensitively and quickly react with an adaptive response. To what extent this response may have a neuroprotective anti-inflammatory effect, or if rather the progression of the disease is amplified in a harmful pro-inflammatory manner, can often only be analyzed incompletely with standard methods. More precise molecular methods must be used to map the entire spectrum of microglial phenotypes and their functional consequences.

### 3.3. Lymphocyte Cells

T lymphocytes establish the connection from the peripheral immune system to the immune system of the CNS, and are of importance for the modulation of neuroinflammation in conjunction with CNS microglia. Together with B lymphocytes, T lymphocytes build the adaptive immune system. After maturing in the thymus, T lymphocytes circulate continuously in the blood until they are activated by antigen-presenting cells, such as dendritic cells, B cells, or microglia. A rough distinction is made between two classes, the CD8^+^ T cells and the CD4^+^ T cells. CD8^+^ T cells, also known as cytotoxic T cells, are activated by the MHCI complex and predominantly induce apoptosis of damaged or infected cells. CD4^+^ T-helper cells, which are activated by the MHCII complex, ensure a regulation of the immune response. The proteins of the MHC complex are a central element in immune regulation and present antigenic structures that can activate T lymphocytes [61].

In the midbrain of mice overexpressing aSyn, CD3^+^ T cells were detected in significant numbers. In an additional analysis of the entire brain, similar amounts of CD4^+^ and CD8^+^ cells were detected [62]. Local overexpression of aSyn in midbrain neurons by viral vectors led to dopaminergic neurodegeneration [30], which was shown to be associated with a locally significantly increased proliferation of T and B lymphocytes being accompanied with microglial activation [49]. The maturation of T lymphocytes similarly seems to depend on the expression of alpha-synuclein, because aSyn knockout mice showed significantly reduced numbers of CD4^+^, CD8^+^, and even double CD4^+^/CD8^+^ negative cells compared to controls [63].

Looking at these data, it can be stated that the lymphocyte cell population is importantly involved in the modulation of neuroinflammation in PD models through its interaction with the innate immune system. 

## 4. Alpha-Synuclein Pathology and Neuroinflammation in Human PD

In the human PD brain, perikaryal Lewy bodies represent the neuropathological hallmark and contain aggregated aSyn as a main component. Lewy neurites represent filamentous neuritic inclusions of degenerating dopaminergic neurons [64,65,66]. In familial PD, various point mutations in aSyn (A30P, E46K, H50Q, G51D, A53E, and A53T) have been identified, but also rare gene duplications or triplications exist which cause additional autosomal-dominant or -recessive forms of PD [67]. The pathological findings on neuroinflammation in human PD are extensive but also heterogeneous. This may not least be due to the fact that the clinical diagnosis of PD continues to be associated with uncertainties [68]. For this reason, it has to be assumed that the human data collected so far contain a proportion of patients who have been incorrectly diagnosed, and therefore findings on neuroinflammation may be more diverse. Another ongoing challenge is the pre-analytical processing of biosamples, as well as the analytical and clinical procedures, which need to be standardized in the best possible way and should be performed with a harmonized protocol in multicentric cohorts [69,70].

Initial analyzes of the neuroinflammation of PD were already performed 100 years ago on brain post mortem tissue [71]. In the neuropathological studies, the degeneration of dopaminergic nigral neurons and Lewy-body pathology were in focus first [72], but soon after the involvement of extra-nigral brain regions [73] and glial pathology was appreciated [74].

### 4.1. Astroglia

There are indications of astrocytic involvement in PD since very early investigations [74,75], although their impact varied in severity with different analytical techniques [76]. The topographical distribution pattern of aSyn immunoreactive astrocytes appears to parallel the formation of neuronal aSyn inclusion bodies in cortical neurons. This could depend on the proximity to terminal axons of affected cortico-striatal or cortico-thalamic neurons [77]. Compared to other brain regions, the astrocyte density in the SN is rather low, which could also explain the increased vulnerability of dopaminergic neurons to cellular stressors [78]. Astrocytes appear in two morphological phenotypes as protoplasmic astrocytes in the gray matter or as fibrous astrocytes in the white matter. Protoplasmic astrocytes surround the neuronal cell bodies and synapses, while fibrous astrocytes surround Ranvier’s rings and oligodendroglia [32].

The function of astroglia is strongly influenced by microglia due to their close (patho)physiological interactions. Recently, in analogy to the classification of microglial cells into an M1 and M2 phenotype, a subdivision of the astrocytes into a destructive A1 and protective A2 phenotype has been postulated [79]. An astrocytic A1 phenotype was shown to be induced by classically activated microglia via secretion of the cytokines IL-1α, TNF-α, and complement component 1q (C1q), thereby losing the ability to promote neuronal survival and outgrowth or to support synaptogenesis. Rather, in the presence of this cell type, the probability for cell death of neurons and oligodendrocytes is increased. The histological analysis of astrocytes in the SN in human PD showed a significantly increased population of destructive A1 cells, so that a close connection with dopaminergic cell pathology could be established [79]. In knowledge of the strong activating stimulus of aSyn for microglia, it seems very likely that such a context promotes the A1 phenotype. In an own analysis, we detected increases of astrocytic cell numbers in the striatum of PD patients, which was also associated with increased expression of the protein ROCK2, a regeneration-inhibiting protein. This alteration was not found to the same extent in the SN. The striatal expression of regeneration inhibitory factors may thus indicate another cause for why dopaminergic axonal pathology develops [80]. 

The expression of aSyn in astrocytes in PD human brains is rather low, but it has been found in various aggregation states [77,81]. ASyn can accumulate in astrocytes, predominantly in non-fibrillar forms. In human neuropathological investigations, mostly fibrous astrocytes were shown to be affected [82]. They were located not only in the SN, but also in regions such as the striatum and dorsal thalamus, where dopaminergic terminals reside [77]. This indicates again a possibly harmful constellation for dopaminergic neurotransmission. 

### 4.2. Microglia

The microglial cell population probably plays the most important role in neuroinflammation in human PD, but also in other neurodegenerative diseases [83]. It is found in an altered state both in the early stages and in the progressive course of the disease. Compared to healthy controls, a microglial cell number increase was shown in several post mortem studies on PD brains. Microglia were primarily located to the SN as identified by immunohistochemical detection of the complement receptor CR3/43, ferritin, or HLA-DR [76,84]. In the case of MHCII-positive microglia, an increased aSyn deposition was additionally found [85]. Other brain regions, such as putamen, hippocampus, transentorhinal cortex, or cingulus gyrus, also exhibited increased microglial cell populations [86]. In our own study, we detected a striatal increase in the number of ED1-positive microglial cells compared to age-matched healthy controls. This was accompanied with an increased expression of the regeneration-inhibitory protein ROCK2 and astrogliosis, indicating that there may be a microglial-astroglial interplay that impairs dopaminergic regenerative function [80]. The morphological phenotype of human microglia ranges from the ramified putative “resting state” to the “activated” or “phagocytotic” variant [87]. However, in our opinion this does not permit drawing a definite inference to their (patho)physiological function. Activated microglia express enzymes, such as iNOS, NADPH oxidase, COX, or myeloperoxidase, which are oxidative stressors that can potentiate inflammatory processes. An increased expression of these enzymes can be found in post mortem analyzes in the SN of PD patients [88,89]. Interestingly, microglia-associated pro-inflammatory cytokines, such as TNF-α, IL-1β, and IL-2, IL-10, or CRP, may be significantly increased in PD peripheral blood analyzes [90].

Much attention is currently being paid to nuclear medicine methods that label microglia in PD patients in vivo with positron emission tomography (PET) technology. The application of the marker [^11^C]*(R)*-PK11195 for activated microglia to drug naïve early disease-stage PD patients showed a significantly higher density of microglia in the midbrain and putamen on the clinically more affected side. This also correlated with a reduced activity of dopamine transporter ligand [^11^C]CFT and motor function [91,92]. Increased accumulation of microglia, also labelled with [^11^C]*(R)*-PK11195, has recently been demonstrated, even in patients with REM sleep behavior disorder (RBD)—a high-risk prodromal feature of PD. In this study, a left-sided increased nigral accumulation of [^11^C]*(R)*-PK11195 correlated with a decreased left-sided putaminal signal of the dopaminergic marker [^18^F]-DOPA [93]. 

However, these findings have to be interpreted with caution, because [^11^C]*(R)*-PK11195 or other TSPO ligands are not exclusively specific for microglial cells, as recent studies have demonstrated. It is known that microglial activation in response to various stimuli is associated with an upregulation of its mitochondrial translocator protein-18 kDa (TSPO) [94], which is also the case in various neurodegenerative diseases, including PD [95]. But TSPO is also involved in several physiological processes, such as steroidogenesis, mitochondrial oxidation, cell growth, and differentiation, and thus is not a discriminating factor to differentiate microglial phenotypes. Furthermore, a common single nucleotide polymorphism (rs6971) in exon 4 of the *TSPO* gene affects the ligand-binding affinity [95]. Importantly, TSPO can also be expressed on activated astroglia [96]. Novel second-generation TSPO ligands labeled with the short-lived positron-emitter isotopes ^11^C and ^18^F like [^11^C]-PBR06 or [^18^F]-DPA-714 enable the visualization of TSPO expression with improved signal to nose ratios and are currently extensively studied in human clinical studies. Here, quantification of data is a complex task, because microglia are distributed ubiquitously throughout the entire brain, and premorbid disease may occur. Advanced methods using cluster analysis could be performed with dynamic PET scans of each individual to determine suitable reference regions [95]. The identification of new targets that specify either pro- inflammatory or anti-inflammatory microglial phenotypes will provide further information on the role of these immune cells and provide a mechanistic rationale for the development of more effective neuroprotective drugs, including antibody-based therapies. A novel microglial marker candidate is the macrophage colony-stimulating factor 1 receptor (CSF1R), which was successfully targeted with a positron-emitting, high-affinity ligand [^11^C]-CPPC. It showed specific and elevated uptake in a murine model of AD and in post mortem brain tissue of AD patients [97].

### 4.3. Lymphocyte Cells

The appearance of lymphocyte cells has been primarily demonstrated in post mortem SN analyzes of PD patients [84,85,98,99]. Interestingly, in the peripheral blood of PD patients, significant differences were found in the abundance of lymphocyte subpopulations, which indicated altered adaptive immunity and were associated with motor dysfunction [100]. In other studies, the ratio of CD4^+^ to CD8^+^ cells decreased, the number of regulatory CD4^+^/CD25^+^ T cells was reduced, or the total number of CD4^+^ T cells and CD19^+^ B cells were decreased [101,102]. This indicates that in PD patients, possibly due to aSyn pathology, maturation deficits or dysregulated populations of T lymphocytes develop, as has also been observed in animal studies [63]. 

In several genetic examinations, an association of PD with the individual configuration of alleles of the MHC complex was found [103,104]. To clarify the question of whether PD is associated with T-cell recognition of aSyn epitopes presented by specific MHC alleles, Sulzer et al. examined PD patients and age-matched controls. After stimulation of human PBMC with IFN-γ or IL-5 in one or two-thirds of the patients, respectively, a reaction to aSyn peptides of the Y39-near N-terminal or S129-related C-terminal region was found. This response was mediated by CD4^+^ or CD8^+^ T cells and was especially robust with defined subsets of MHC alleles [105]. Approximately 40% of the PD patients in the study exhibited immune responses to aSyn epitopes, which could reflect variations in disease progression or environmental factors. In classic autoimmune disorders, such as type-1 diabetes or rheumatoid arthritis, a similar percentage of patients display these responses [106]. Here, the MHC class II response may precede MHC class I, and it was noted in the Sulzer manuscript that exposing microglia to aSyn activated MHC class I expression by dopamine neurons [105]. 

These data demonstrate that aSyn protein fragments can induce T cell-mediated immune responses in PD patients and raise the suspicion that PD could be an autoimmune disorder. To what extent the risk of progression of disease in PD individuals with certain MHC allele configurations may be increased must be investigated in further studies.

From the overall point of view, it becomes clear that there is a relevant neuroinflammatory component also in human PD neuropathology, which is documented in post mortem analyzes as well as in in vivo investigations at different time points during the disease course [74,93,107]. Unfortunately, longitudinal interindividual or even intraindividual analyzes are missing or are only available for small patient numbers and periods of time in which no significant alterations have been observed [107]. 

## 5. Immunotherapies to Target Alpha-Synuclein

As shown in the previous sections, aSyn pathology is a central hallmark of PD. Both in animal experiments and in human studies, it has been shown that accompanying neuroinflammatory processes involving astroglia, microglia, and T cells play a role for disease development. Therapeutic strategies with direct targeting of single or multiple aspects of cellular neuroinflammation are currently under investigation and are described elsewhere [44,108]. Another therapeutic approach is to primarily focus on aSyn as the causative substrate of this pathology. The main goal here is to lower the burden of extracellular aSyn in the brain, and as a consequence reduce neuroinflammation. Until now, passive immunization with administration of antibodies directed against aSyn, seems to be one of the most promising strategies to accomplish this objective [109]. In the following section, an overview of the most important recent results with the passive aSyn immunotherapies are presented.

### 5.1. Preclinical Data

Passive immunization in PD is performed by an anti-aSyn antibody transfer into the CNS. The two primary targets for monoclonal antibodies are the aSyn C-terminal region [110,111,112] and the aSyn N-terminal region [113,114]. In the following section, only data with aSyn transgenic mice will be discussed because toxin-based PD mouse models have not been examined for this therapeutic approach.

In 2011, a monoclonal antibody that targets the C-terminus of aSyn (9E4) was administered for a period of 6 months with once weekly intravenous injections of a dose of 10 mg/kg body weight to a transgenic mouse model of PD. The mice exhibited an improved behavioral response in the water maze and showed less aggregation of calpain-cleaved aSyn in synapses and axons of both cortical but also hippocampal neurons [111]. One year later, a second group applied a different C-terminal antibody (AB274) to the identical mouse model with once weekly injections intraperitoneally. In their study, behavioral motor deficits improved too, and reduced neurodegeneration in the passively immunized group could be demonstrated after 4 weeks. Interestingly, an enhanced localization of aSyn and AB274 in microglial cells was found. It could be shown on a microglial cell line that the uptake of the AB274/aSyn complex through microglial cells was mediated via the Fcγ receptor and directed into lysosomal compartments [110]. In a third study with anti-aSyn antibodies directed against the C-terminally, the compounds 1H7, 5C1, and 5D12 were administered once weekly for a total of six weeks in another transgenic PD mouse model. Here, the behavioral performance was improved and the expression of aSyn in mouse cortex and striatum after application of 1H7 and 5C1was significantly reduced. This was accompanied by a less severe axonal pathology [112].

With regards to anti-aSyn antibodies that are N-terminally directed, Weihofen et al. reported recently on the identification, binding characteristics, and efficacy in mouse models of PD with the human-derived aSyn antibody (BIIB054) [115]. In three different experiments with intracerebrally inoculated preformed aSyn fibrils, BIIB054 treatment for 3 months or longer was shown to be able to attenuate the spreading of aSyn pathology, rescue motor impairments, and reduce loss of dopamine transporter density in dopaminergic striatum terminals [115]. In another study, intraperitoneal administration of an anti-aSyn antibody (AB1) two weekly for a total of three months resulted in a moderate but insignificant improvement in behavioral parameters. Nigral dopaminergic neurons were robustly protected against cellular loss and the general expression of aSyn, as well as the activation of microglia was found to be decreased in this brain region [113]. A treatment of mice that had been intrastriatally injected with aSyn pre-synthetic fibrils with an anti-aSyn N-terminal antibody (Syn303) was undertaken in order to find a more specific antibody design for misfolded aSyn. This antibody was applied weekly for a period of up to half a year and successfully inhibited the spread of aSyn aggregates, as well as improved behavioral deficits and loss of dopaminergic cells [114]. 

In additional studies on transgenic PD mice, these were either weekly immunized for more than three months with antibodies (Syn-F1, Syn-O1, and Syn-O4) directed specifically against oligomeric and fibrillary aSyn but not against monomeric aSyn [116], or received weekly passive immunizations for a total of 14 weeks with a monoclonal antibody that targeted oligomeric/protofibrillary forms of aSyn (mAb47 antibody) [117]. Both approaches led to significant decreases in the accumulation of pathogen aSyn fibrils and improved behavioral performance measures. Passive immunization of mice overexpressing human aSyn in oligodendrocytes (PLP-aSyn mice) resulted in a reduction of total aSyn in the hippocampus and less intracellular accumulation of aggregated aSyn, particularly in the spinal cord. This was accompanied by a reduced amount of activated microglial cells [118]. If non-transgenic, aSyn knock-out, or aSyn transgenic mice received unilateral intra-cerebral injections with an aSyn-expressing viral vector construct, the axonal accumulation of aSyn in the contralateral side could be reduced with a three-month systemic administration of a monoclonal anti-aSyn antibody (1H7, recognizing amino acids 91–99) [119].

The evaluation of neuroinflammatory changes in the above-mentioned animal studies after passive immunization differs greatly in the scope of the analyzes. Overall, the regulation of microglia is a common parameter, which is often interpreted to have been shifted to a more beneficial level. The function of astrocytes is only analyzed in a few studies and T cell responses are not described. An overview is shown in Table 1. 

Apart from antibody-based therapeutic strategies to alleviate aSyn toxicity and promote its clearance, another option is to employ small molecules. Nilotinib is a c-Abl tyrosine kinase inhibitor that is used in the treatment of chronic myelogenous leukemia. It has recently been shown to be able to both alleviate c-Abl associated aSyn aggregation and impaired autophagy, as well as protect dopaminergic neurons in mouse models of PD [120]. Importantly, the level and activity of c-Abl can be elevated in human PD in both the substantia nigra and striatum [121], so that the potential of c-Abl inhibitors as a symptomatic and causative treatment is currently under evaluation in a drug repurposing approach [122].

In view of these data, it can be stated that the anti-aSyn-directed immunotherapies are effective in animal models—whether directed against the C-terminus or the N-terminus of aSyn—and clearly demonstrate “target engagement”, reduce the lesion burden of aSyn (mostly in a specific manner), can to some extent probably alleviate “αactivation” of cellular inflammatory responses, and achieve improvements in motor behavior. These promising findings were strong arguments to proceed to clinical trials in humans.

### 5.2. Human Clinical Data

Various human clinical trials using anti-aSyn antibodies have been performed in a phase I clinical design with healthy volunteers. Overall, they have shown a good safety profile and tolerability. More recently, large phase II clinical trials have been launched and will soon provide data on safety, tolerability and to some extent on treatment effects in PD patients. 

PRX002 was the first passive therapeutic immunization using an anti-aSyn directed antibody that was developed by Prothena. It is a C-terminally directed humanized IgG1 monoclonal antibody and has been studied in animal models before, as mentioned above [111]. In a phase I study with a single ascending dose, PRX002 show good tolerability and safety with intravenous infusions of 0.3, 1.0, 3.0, 10, or 30 mg/kg. After a single infusion of 30 mg/kg, serum PRX002 antibody levels were shown to be increased up to 578 μg/mL. Within one hour from PRX002 administration, there was a significant reduction of free aSyn in serum, which was dose-dependent. Interestingly, levels of total aSyn that comprise free and bound aSyn increased in a dose-dependent way. This was presumably due to an expected change in kinetics after the binding of antibodies. The average terminal half-life across all doses was 18.2 days [123]. 

This trial was followed by another phase I study with multiple ascending-doses in patients with mild or moderate idiopathic PD (HY stages 1–3). These received three intravenous infusions of PRX002 (0.3 mg/kg, 1.0 mg/kg, 3.0 mg/kg, 10 mg/kg, 30 mg/kg, or 60 mg/kg) or placebo at intervals of 4 weeks. Data on tolerability and safety were favorable. Serum levels of PRX002 increased dose proportionally, whereas serum free-to-total serum levels were strongly reduced. The antibody mean terminal elimination half-life was comparable for all doses, approximating 10.2 days. The mean CSF concentration of the study compound increased dose dependently and was approximately 0.3% relative to serum across all dose cohorts. However, it was not possible to demonstrate any alteration in the CSF aSyn level, probably due to the relatively weak affinity of the antibody for the monomeric forms, as opposed to aSyn aggregates, which are much less prominent in the CSF [124]. In 2017, Prothena and Roche launched a multinational phase II study of PRX002/RO7046015 in patients with newly diagnosed PD (PASADENA Study, ClinicalTrials identifier NCT03100149). 

BIIB054 is a fully human anti-aSyn IgG1 monoclonal antibody directed at the N-terminus of aSyn. It has been developed by Neurimmune, a Swiss biotech company, and was originally isolated from B cell lines that had been generated from neurologically healthy individuals. It is very selective for the aggregated form of human aSyn that has been detected in tissue sections from PD and DLB patients [125]. A good safety and tolerance were recently demonstrated at single doses with up to 90 mg/kg in a phase I study in healthy volunteers. The BIIB054 serum half-life was 28 days, its CSF concentrations obtained were 0.2% of those observed in plasma. In PD patients, single doses of BIIB054 up to 45 mg/kg were well tolerated, the CSF:plasma ratio was 0.4%, and the pharmacokinetic profile was comparable to that observed in healthy volunteers [126]. Currently, BIIB054 is being evaluated in a multinational phase II study with patients with newly diagnosed PD (SPARK Study, ClinicalTrials identifier NCT03318523).

The MEDI1341 monoclonal and the BAN0805 monoclonal anti-aSyn antibodies are two more compounds which are currently being planned to be tested in single-center studies of single ascending intravenous doses in phase I clinical trials. MEDI1341 appears to have only a reduced immune effector function and is now developed in collaboration of AstraZeneca and Takeda (ClinicalTrials.gov identifier NCT03272165). BAN0805 is more specifically targeting aSyn oligomers as well as protofibrils, and is being developed in a collaboration between Abbvie and Bioarctic [127]. 

These clinical studies are all carried out at a very high pace of development and take place at a high medical technology level. Unfortunately, no data on the modulation of neuroinflammatory parameters, e.g., in blood, serum, or other compartments, are publicly available.

## 6. Conclusions

It is undisputed that neuroinflammatory processes are significantly associated with the neuropathological progression and clinical course of PD. Through very elaborate studies on patients, but also in animal models of the disease, core pathomechanisms have been identified, which apart from the damage to neuronal cells also affect glial cells and are regulated by neuroimmunological interactions. This opens up a large window for neuroimmunological interventions with the ultimate goal to modify the course of disease. 

Although more focused interventions with targeted regulation of the function of astrocytes, microglia, or T lymphocytes will become possible in the future, the knowledge of the multicellular spreading PD neuropathology seems to make a rather broad and systemic approach meaningful. Therefore, the reduction of CNS aSyn by targeted antibody-based technologies is a valid tool. In future human clinical studies, the target engagement and effectiveness of anti-aSyn antibodies to reduce aSyn burden in the CNS should be very precisely monitored. This could be achieved by detection of CSF aSyn with enzyme-linked sandwich-type immunosorbent assays (ELISA) or even more precise analyzes for its aggregated states with Protein Misfolding Cyclic Amplification (PMCA) or Real-Time Quaking-Induced Conversion (RT-QuIC) [128]. A non-invasive in vivo analysis of the brain aSyn load would be possible via nuclear medicine PET or SPECT methodology, but this is currently not established [129]. 

In contrast, the PET technology for specific labeling of microglial cells has improved and could be integrated with the use of second-generation microglial TSPO tracers [130]. First-generation tracers have been shown to perform well already in prodromal stages of PD [93] and have also been used as cellular readout in microglia-targeted therapeutic approaches [131]. One challenge remains to quantify the extent of CNS neuroinflammation as precisely as possible. In addition to the most specific markers possible, a comprehensive assessment must map the various cell populations and also represent individual phenotypes. With regard to anti-aSyn immunotherapies, the characterization and quantification of aSyn-dependent immune effects should be a main goal, especially during the course of therapy. Without such measures, the assessment of a change in neuroinflammation under passive immunotherapy remains limited. 

From a practical point of view, an improved applicability of antibodies with subcutaneous injections would facilitate their use in clinical studies even more. Safety and tolerability issues will always remain primary goals for the development of novel agents. If these requirements can be met, it will only be a matter of time before an effective method of anti-aSyn antibody-based therapy is developed that effectively modulates cellular neuroinflammation and also achieves clinically relevant improvements in PD patients.

## Figures and Tables

**Table 1:** Passive immunization studies in Parkinson’s disease animal models and therapeutic responses.

Antibody	Target Site of aSyn	Behavioural Alterations	Cellular Responses of				Reference
			Neurons	Astroglia	Microglia	T Cells	
9E4	C-terminal	Cylinder Test ↑	DA cell loss (30%) ↓	-	-	-	[111]
Ab274	C-terminal	Pole test and total activity ↑	NeuN cell loss ↓	Astroglial aSyn accumulation ↓	Microgliosis ↓ Increased aSyn clearance by microglia	-	[110]
1H7 5C1	C-terminal	Probe/transversal round beam test ↑	DA cell loss (35%) ↓	Astrogliosis ↓	Microgliosis ↓	-	[112]
AB1 AB2	N-terminal Central region	Cylinder test ↑	DA and NeuN cell loss ↓	-	Microgliosis ↓	-	[113]
Syn303	N-terminal	Wirehang ↑	Blocked aSyn spreading DA cell loss ↓	-	-	-	[114]
BIIB054	N-terminal	Wirehang ↑	Loss of dopamine transporter density in the SN ↓	No change	No change	-	[115]
mAB47	Protofibrils	-	aSyn protofibrils in spinal cord ↓	No change	No change	-	[117]
Syn-O1 Syn-O4 Syn-F1	Oligomers Oligomers Fibrils	Total activity in open field test ↑	aSyn accumulation ↓ NeuN cell loss ↓	Astrogliosis in hippocampus ↓ (O1,O4)	Microgliosis in hippocampus ↓ (O1,O4)	-	[116]

## Abbreviations

AP	Activator protein
aSyn	Alpha-Synuclein
CD	Cluster of differentiation
CIITA	Class II transactivator
CNS	Central nervous system
COX	Cyclooxygenase
CR	Complement receptor
CRP	C-reactive protein
CSF	Cerebrospinal fluid
DA	Dopamine
DOPA	Dihydroxyphenylalanine
ELISA	Enzyme-linked sandwich-type immunosorbent assays
HLA-DR	Human leukocyte antigen—DR isotype
IFN	Interferon
Ig	Immunoglobulin
IL	Interleukin
iNOS	Inducible nitric oxide synthase
MHC	Major histocompatibility complex
NAC	Non-amyloid-beta component
NADPH	Nicotinamide adenine dinucleotide phosphate
NFκB	Nuclear factor ‘kappa-light-chain-enhancer’ of activated B-cells
NMDA receptor	N-methyl-D-aspartate receptor
NO	Nitrogen monoxide
Nrf2	Nuclear factor erythroid 2-related factor 2
NSF	N-ethylmaleimide sensitive factor
PBMC	Peripheral blood mononuclear cell
PD	Parkinson’s disease
PET	Positron emission tomography
PMCA	Protein misfolding cyclic amplification
PNS	Peripheral nervous system
RNA	Ribonucleic acid
ROCK2	Rho associated coiled-coil containing protein kinase 2
ROS	Reactive oxygen species
RT-QuIC	Real-time quaking-induced conversion
SN	Substantia nigra
SNARE	Soluble N-ethylmaleimide sensitive factor attachment receptor
SPECT	Single photon emission computed tomography
TGF	Transforming growth factor
TLR	Toll-like receptor
TNF	Tumor necrosis factor
TNT	Tunneling nanotubes
TSPO	Translocator protein-18 kDa
